# The ethical anatomy of payment for research participants

**DOI:** 10.1007/s11019-022-10092-1

**Published:** 2022-05-24

**Authors:** Joanna Różyńska

**Affiliations:** Center for Bioethics and Biolaw, Faculty of Philosophy, University of Warsaw, Krakowskie Przedmiescie 3, 00-047 Warsaw, Poland

**Keywords:** Research ethics, Payment for research participants, Social beneficence, Autonomy, Justice/fairness, Undue inducement, Exploitation

## Abstract

In contrast to most publications on the ethics of paying research subjects, which start by identifying and analyzing major ethical concerns raised by the practice (in particular, risks of undue inducement and exploitation) and end with a set of—more or less well-justified—ethical recommendations for using payment schemes immune to these problems, this paper offers a systematic, principle-based ethical analysis of the practice. It argues that researchers have a *prima facie* moral obligation to offer payment to research subjects, which stems from the principle of social beneficence. This principle constitutes an ethical “spine” of the practice. Other ethical principles of research ethics (respect for autonomy, individual beneficence, and justice/fairness) make up an ethical “skeleton” of morally sound payment schemes by providing additional moral reasons for offering participants (1) recompense for reasonable expenses; and (2a) remuneration conceptualized as a reward for their valuable contribution, provided (i) it meets standards of equality, adequacy and non-exploitation, and (ii) it is not overly attractive (i.e., it does not constitute undue inducement for participation or retention, and does not encourage deceptive behaviors); or (2b) remuneration conceptualized as a market-driven price, provided (i) it is necessary and designed to help the study achieve its social and scientific goals, (ii) it does not reinforce wider social injustices and inequalities; (iii) it meets the requirement of non-exploitation; and (iv) it is not overly attractive. The principle of justice provides a strong ethical reason for not offering recompenses for lost wages (or loss of other reasonably expected profits).

What are the ethical principles or values which constitute an ethical rationale for paying research subjects? Do those create a moral obligation to pay individuals for participation in biomedical research, or rather a mere justification for its acceptability? What other ethical principles and values shape the payment practice, and how? Surprisingly, these questions have been rarely the subject of in-depth discussions in the literature. Instead of exploring the ethical foundations of payments systematically, scholars and public-policy makers rather focus on payment-related ethical concerns, in particular of undue inducement and exploitation, and—from this perspective—recommend or discourage certain forms, schedules and timings of payment commonly used in research practice (e.g., Macklin 1981; Dickert and Grady 1999; Grady 2001, 2005; Gelinas et al. 2018; Resnik 2015, 2019; Largent and Lynch 2017a, 2017b). As a consequence—while there is a growing consensus that an ethically sound payment scheme should avoid both excessive payment and underpayment, and it should include, at least, reimbursement of reasonable expenses and compensation for *some* contributions made by research subjects—there is no generally accepted view on whether a payment to research subjects (as such or of a certain kind) is a moral obligation (Council for International Organizations of Medical Sciences 2016), merely an “acceptable practice” (Food and Drug Administration 2018), “ethically discretionary” activity (Persad et al. 2019, p. 319), or just a “necessary evil”. Equally, there is no common view on what constitutes an ethical source of this purported obligation or acceptability of payment (as such or of a certain kind), and which ethical reasons lie behind different payment categories and schemes.

This paper aims at clarifying these issues. It presumes that any discussion on ethically sound payment practice should be preceded by a clear statement of ethical reasons for paying research participants, their deontic nature and mutual relations. Without full understanding of the ethical anatomy of payment, it is impossible to determine what we owe, if anything, to research subjects—what for, and how much research participants should be paid.

## Preliminary terminological remarks

Paying research subjects for their participation in biomedical studies is an increasingly common practice across different types of research involving healthy volunteers and patients (Grady et al. 2005; Largent and Lynch 2017a). Nevertheless, the payment continues to raise numerous conceptual, ethical and practical controversies among bioethicists, investigators, research ethics committees/institutional review boards (RECs/IRBs), and other members of the research community. Although prominent international guidelines and national regulations call attention to the crucial moral issues that payment raises (in particular, the risk of undue influence), they offer little substantive guidance on how to pay research subjects in an ethical way, and if they do so, they often provide contradictory advice. For instance, the World Medical Association Declaration of Helsinki (2013) does not address payment for research participation directly. It only mentions that information on “incentives for subjects” must be contained in the study protocol (par. 22). Also, the U.S. “Common rule” (Department of Health and Human Services 2018) and the European Union “Clinical Trial Regulation” (2014) offer very limited guidance on payment for participants. All these regulatory lacunas and contradictions are reflected in considerable variation in local payment policies and practices around the world (Dickert et al. 2002; Grady et al. 2005; Fry et al. 2005; Ndebele et al. 2008; Pasqualetti et al. 2010; Roche et al. 2013; Largent and Lynch 2017a).

One of the factors which adds to this confusion is the diversity and ambiguity of terminology used in the literature and guidelines on research payment. For example, the *International ethical guidelines for health-related research involving humans* of the Council for International Organizations of Medical Sciences (CIOMS) make a distinction between “reimbursement” for reasonable direct costs incurred by research subjects and “compensation” for the time spent and other inconveniences resulting from study participation (2016, Guideline 13 and Commentary). The *Guideline for good clinical practice* issued by the International Conference on Harmonisation of Technical Requirements for Registration of Pharmaceuticals for Human Use speaks of “payments and compensation” (2016, par. 3.1.2., 3.1.8., 3.1.9.) with no further explanation. The U.S. Food and Drug Administration *Guidance for institutional review board and clinical investigators: payment and reimbursement to research subjects* (2018) makes a distinction between “reimbursement” for direct costs and other “payment” for participation, which it considered to be more ethically challenging as in some cases it may constitute an undue influence to prospective subjects. The Council of Europe’s *Additional Protocol to the Convention on human rights and biomedicine, concerning biomedical research* mentions „payments and rewards” without any additional differentiations, in the Appendix containing a template of an information sheet for the ethics committee (2005a). The term is “unpacked” in the Explanatory Report to Article 12 of the Additional Protocol dealing with undue inducement which refers to “compensation” for burdens and inconveniences and “reimbursement” for expenses and financial losses (Council of Europe 2005b, sec. 64). Australian guidelines on *Payment of participation in research: information for researchers, HRECs and other ethics review bodies* issued by the National Health and Medical Research Council (2019) divide “payment” for participants in four analytical categories: “reimbursement” for any research-related expenses; “compensation” for any documented financial losses resulting from participation in research, including loss of wages, or from an injury suffered as a direct consequence of participation; “remuneration” paid to participants in recognition of their service for the time spent and other inconveniences resulting from participation; and “incentive or inducement” provided to individuals simply to encourage their enrolment or continuation in research. In the United Kingdom, the NHS Health Research Authority guidance (2014) follows terminology developed by the Nuffield Council on Bioethics. The Council, in its report *Human bodies: donation for medicine and research* (2011) distinguishes three forms of payment: (i) “recompense” offered in recognition of losses incurred which may take a form of “reimbursement” of direct financial expenses, lost earnings, or “compensation” for non-financial losses, such as time, inconvenience and discomfort; (ii) “reward” defined as a “material advantaged gained by a person …that goes beyond ‘decompensating’ the person for losses they incurred”; “reward” becomes “remuneration” when calculated as a wage or equivalent”; and (iii) “purchase”—money given in exchange of a “thing” (e.g., biological material for research) (Nuffield Council on Bioethics 2011, p. 2).

In order to bring an end to this terminological (and conceptual) chaos, in this analysis, “payment” is used as an overarching term that encompasses all forms of financial and in-kind support provided to research participants.^1^ It covers two sub-categories, which will be referred to as “recompense” and “remuneration”. These categories are distinguished by a different impact each of them has on the participant’s economic position as evaluated *ex post*.

The term “recompense” stands for any payment that entails no net benefit to recipients. Recompense amends to research participants for financial and non-financial losses or injuries resulting from their participation in research. Thus, this type of payment does not constitute a gain or profit, but merely covers—understood in broad terms—costs of the participation. Recompense may include three sub-categories of payments: (i) reimbursement of direct financial expenses incurred as a result of participation in research (e.g. costs of travel, accommodation, meals, childcare); (ii) compensation for indirect financial expenses, i.e., losses, that arise from participation in research (e.g. loss of wage in result of taking unpaid leave from work); and (iii) compensation for financial and non-financial losses resulting from injuries suffered as a direct consequence of participation in research.

“Remuneration” refers to any payment provided to individuals for their service as research participants, which exceeds expenses, losses or injuries experienced by participants as a result of their participation, and brings net benefit (gain, profit) for recipients. The remuneration for research participation (same as for “regular work”) may be understood either as a reward given in recognition of and as an appreciation for participants’ valuable contribution; or as a price—money given in direct, market-driven exchange for a service provided by participants (cf. Moriarty 2020; Różyńska 2021). If the remuneration is understood as a reward, the payment should be adequate to the desert, i.e., to the value of participants’ contribution. The latter value necessary depends on various factors, including (i) time allotted to research; (ii) efforts or types of services rendered (e.g., performing psychological or physical tests, taking drugs or using device as instructed, conducting self-monitoring or gathering other research-relevant information); (iii) discomfort, burdens or inconveniences associated with participation (e.g., stress, pain, suffering, but also burdens related to sticking to a dietary regime or inconveniences caused by the expected lifestyle changes); (iv) the level of risk involved in research, and (v) special/unique value of the input of the specific subject (e.g. due to rarity of a disease suffered by the person or her genetic make-up). If remuneration is considered as a price for participation, the amount of payment is determined by market forces—supply and demand, and it does not have to be proportionate to the value of the subjects’ contribution. In literature and guidelines, the latter way of thinking about payment if often concealed under the concept of “incentive” or “inducement”, although—as it will be explained later—these labels are far from being adequate and should be abandoned.

The above typology of payments for research participation is preferable over other schemes proposed in the literature and regulatory documents at least for three reasons. First, it is built upon one clearly defined, objective and disjoint divisional criterion, thereby avoiding a mistake of mixing entirely different criteria in one scheme. Such a mistake affects all typologies of research payments which, alongside recompenses and remunerations, distinguish “incentives” or “inducements” payments. This mistake originates from the fact that the former two categories of payments are defined by the payment impact on the subject baseline economic position, the latter is based on the researcher’s intention. Secondly, by rejecting the term”compensation”, the proposed typology avoids conceptual and normative confusion stemming from ambiguity of this notion in legal terminology, where it stands for both money received in return for services rendered, especially salaries or wages, and for payment of damages for loss or injury (Różyńska 2021). The ambiguity undermines the conceptual and normative value of highly popular payment scheme advocated by Gelinas, Largent, Lynch, and collaborators (Gelinas et al. 2018; Persad et al. 2019; Lynch et al. 2021; Bierer et al. 2021). Following Christine Grady’s terminology (2005), the authors separate “reimbursement”, “compensation”, and “incentive”. Trapped by the ambiguous language of “compensation”, they frame remuneration as recompense for losses, arguing that “participants' time, as well as their assumption of research-related burden and inconvenience … are critical contributions experienced as losses by participants; they are giving up or accepting unfavorable as a result of participation. Thus, the same justifications applicable to reimbursement applied here” (Lynch et al. 2021, p. 16). At the same time, applying the logic of remuneration, they reject compensating participants based on their opportunity costs, noting that “rather, research compensation is an acknowledgement of participants’ contributions to research, which leads to the conclusion that there generally should be equal pay for equal work” (Lynch et al. 2021, p. 16).

Finally, the advocated typology allows for separating payments form small gifts given at the conclusion of a study (such as chocolates, T-shirts, cups, pens, cinema-tickets) that have minimal market value, serve only as token of appreciation, and have likely zero impact on recruitment (Grady 2005).

Having clarified basic terminological and conceptual issues, we can move on and explore the ethical anatomy of payment for research participants. It would be useful, however, to indicate briefly—from the outset—the scope of the forthcoming analyses.

The paper deals with the widespread practice of paying research participants in exchange for their valuable *service* without determining whether the service should be treated as an unskilled labor, “regular” work, body renting, or a unique *sui generis* endeavor (cf. Lemmens and Elliott 1999, 2001; King Reame 2001; Anderson and Weijer 2002; Elliott 2008; Abadie 2015; Phillips 2011b; Lynch 2014; Różyńska 2018). For the sake of the analysis to come, it is assumed that paid participation in biomedical research is a form of paid bodily services, and it should be “no more worrisome to commodify a person’s labor [bodily service—*JR*] as a research subject than to commodify a person’s labor in other contexts, which happens all the time” (Lynch 2014, p. 159). Therefore, commodification concerns against research payment, raised by some commentators (Macklin 1989; Chambers 2001; Abadie 2010, 2015; Cooper and Waldby 2014; Walker and Fisher 2019) will not be explored here.

Since the paper focuses on the most fundamental ethical basis of payment practice, it is limited to monetary offers (paid in cash, cheques or pre-paid credit cards) as a paradigmatic case of payment, leaving aside all forms of in-kind support. This paper also leaves aside payments to participants unable to give consent, because those raise additional, substantive issues, as well as recompenses for research-related injuries as they would require in-depth legal analysis. The presented analysis applies to research which RECs/IRBs would consider scientifically and ethically sound thus presenting arguments that payment is offered to overshadow or mask some ethical deficiencies of a study project, especially those regarding its risk–benefit profile or criteria of subjects selection. In other words, it considers “research which ethics committee would allow to proceed, were the subject not paid” (Wilkinson and Moore 1997, p. 375).

## Ethical rationale for paying research participants

The main “pragmatic” reason (Largent and Lynch 2017a, p. 77 fn. 22) for offering payment to research participants is to boost recruitment and retention rates. This “efficiency”-driven (Phillips 2011a) rationale is widely acknowledged. It is, however, rarely recognized as having not only practical, but also an ethical facet. Since progress in biomedical science and healthcare is not ethically discretionary, this section argues that neither efficient enrolment nor payment for research subjects, being conductive to the latter, are value-neutral practices. On the contrary, they are ethically grounded in and governed by the principle of social beneficence that calls for maximization of a common good, i.e. good which has two characteristics: it is non-exhaustible (one person’s use does not diminish another’s use) and is beneficial for all or almost all members of a society.^2^

Biomedical research is social practice aimed at generating such a common good—generalizable scientific knowledge that may contribute to the improvement of healthcare interventions and public health measures, thus leading to the advancement of human health (cf. Schaefer et al. 2009; Rhodes 2010; London et al. 2010). Health is highly valued by all individuals and societies primarily “because of what it enables us to do” (Duncan 2010, p. 321). A minimum threshold of physical and mental capacities is necessary (though not sufficient) for an individual to be able to pursue her particular life goals, plans, and projects that express her vision of a good life. Since all liberal and democratic societies share a fundamental moral (and political) commitment to protecting and respecting each person’s right to lead her life in accordance with her personal views of what is valuable in human life, they also share a moral obligation to encourage forms of social collaboration useful in fulfilling basic health needs of their members (London 2003, 2006; Rawls 1971; Nussbaum 2013). This includes the practice of healthcare as well as biomedical research, because the capacity of a society to satisfy its members’ basic health interests is conditional upon progress in biomedical sciences, which in turn is crucially dependent on various types of research, including human research. Thus, societies have a *prima facie* moral obligation to promote the conduct of biomedical research, including research involving human subjects.

Human biomedical research is a complex, collaborative social “enterprise”, involving various institutional and individual stakeholders with various, often conflicting, interests (London et al. 2010; Różyńska 2015). Therefore, in order to fulfill their obligation to promote biomedical research, societies should develop, implement and support normative and institutional mechanisms aimed ensuring the existence, stability and effectiveness of research practice in attaining the common good (London et al. 2010; Resnik 2011; Różyńska 2015). These mechanisms should enhance all stakeholders’ trust and willingness to support and invest in the research enterprise, including prospective research participants without whom biomedical research would not be able to achieve its goals.

Socially valuable human biomedical research is critically dependent on successful enrolment and retention of a sufficient number of appropriate participants, and on their willingness to comply with study procedures and conditions. Failure to recruit or retain participants may lead to invalid or inconclusive research data, it may result in premature termination of a study, increase research costs, and—what is the most important—delay or even hinder scientific progress and the anticipated development of safer and more effective diagnostic, prophylactic and therapeutic interventions (Altman 1980; Halpern et al. 2002; Gul and Ali 2010; Williams et al. 2015; Carlisle et al. 2015). Insufficient or slow recruitment and poor retention rates are, however, a common problem within biomedical research, especially in randomized controlled trials (Salman et al. 2014). A systematic review shows that less than half of trials achieve their original recruitment target goal in time (Campbell et al. 2007; cf. Sully Ben et al. 2013; Parkinson et al. 2019). Additionally, only very small portion of eligible adult oncological patients choose to enroll into trials—depending on a source the portion range from 3 to 5% or 8% (Bell and Balneaves 2015; American Cancer Society Cancer Action Network 2018)—leading to a conclusion that “if the proportion of patients with cancer who agree to participate in clinical trials were to increase from its current 5% to 10%, the usual study completion rate would decrease from around 4 years to 1 year. Another estimate suggests that at least 16 million more individuals are needed to participate in research trials each year” (Schaefer et al. 2009, p. 70).

Many different factors contribute to the low participation and retention rates. Although barriers of structural and clinical character, such as access to trials and eligibility requirements, seems to be the most important, concerns regarding financial costs and benefits linked to research participation also play a significant role in prospective subjects’ decision-making (Friedman et al. 2015; Hamel et al. 2016; American Cancer Society Cancer Action Network 2018; Nipp et al. 2019a, Unger et al. 2013, 2019). Recent statistics published by the Center for Information and Study on Clinical Research Participation show that information about potential costs and their reimbursement as well as information about compensation for time off from work are among the most important factors influencing a decision to participate in research for—respectively—58% and 40% potential subjects (2019). And the prospect of receiving monetary compensation is one of three top reasons (34% mentioning) impacting a decision to enroll into a study (Center for Information and Study on Clinical Research Participation 2019). These data are consistent with results of numerous empirical studies on subjects’ motivations for volunteering, conducted among patients and healthy volunteers. They all indicate that although the payment is not the only reason why people agree to participate in biomedical research, it is definitely one of the top motivates for enrollment, especially among healthy volunteers (Tishler and Bartholomae 2002; Almeida et al. 2007; Abadie 2010; McCann et al. 2010; Stunkel and Grady 2011; Grady et al. 2017; Fisher et al. 2018; Manton et al. 2019).

Thus, although it is rightly stressed in the literature that the exact impact of paying for participation on the rates of research enrollment and retention remains still under-investigated, it is reasonable to assume that payment can make a positive difference in this respect (Dunn and Gordon 2005; Watson and Torgerson 2006; Caldwell et al. 2010; Probstfield and Frye 2011; Treweek et al. 2013; Nipp et al. 2019b; Parkinson et al. 2019). Money may remove participation barriers for those individuals who are unable or unwilling to cover direct costs associated with research, such as costs of traveling, lodging or hiring a babysitter. The payment may attract people who would be otherwise discouraged from the participation by the necessity of taking unpaid leave from work and resulting loss of wages. It may help to make a positive enrollment decision for those, who feel forced by their low social-economic status (SES) to dedicate time and efforts for searching for a job or earning their living, instead of altruistically contributing to the development of science. Additionally, an offer of payment could convince to participation persons who believe that their private or professional time is particularly valuable (for example due to their unique responsibilities, competences, or skills), and, therefore, they should not allot it to alternative causes without an adequate remuneration. In all these cases, payment may enhance recruitment, provided it is designed in a way that targets the underlying barriers, needs, or expectations.

Insofar as these factual assumptions about inducing potential of money are true—and we concede them for the sake of the argument—the principle of social beneficence provides fundamental ethical rationale for paying research subjects. Offering payment to participants is an ethically right and *prima facie* obligatory practice because it increases research recruitment and retention, thereby contributing to the common good produced by the research enterprise. From a broader perspective, “it is useful in fulfilling society’s obligation to meet the essential [health-related] needs of its members” (Ackerman 1989, p. 1). The strength of the obligation to offer payment for participation grows in relation to studies which are urgently needed, e.g., to address acute public-health emergencies (such as a dire pandemic), or when there is strong evidence that without payment recruitment, retention and completion of socially valuable studies would be doomed or severely compromised. The strength of the obligation weakens when—due to study-specific features—payment offers are not needed to secure an adequate number of participants, i.e., where it is reasonable to assume that people will be willing to join the study for non-economic and social reasons, e.g., the prospect of direct or ancillary medical benefits, the wish to make a contribution to medical progress or the health of others, scientific curiosity, interest in the goals of the study, the prospect of making friends or having new experiences (Stunkel and Grady 2011; Grady et al. 2017; Fisher et al. 2018; Manton et al. 2019). Additionally, the obligation to pay research participants may be overridden by justified research budget constraints. If investigators have no money to pay for participation in a socially valuable research project, it is better to allow them to proceed without payment, than obliging them to pay, thereby forcing them to resign from conducting the study (Gelinas et al. 2018).

To sum-up, the payment for research subjects is an incentive or inducement for participation grounded in the principle of social beneficence. This is *expressive verbis* acknowledged by the U.S. Food and Drug Administration (2018) guidance which argues that payment to research subjects is “a recruitment incentive” and by the European Union’s Clinical Trial Regulation (2014) which refers to any payment offered to trial participants as “incentives or financial inducements” (art. 31.1(d); art. 32.1(d), 33(d)). Thus, payment is not, as some scholars and guidelines suggest, a demand of justice or fairness (Gelinas et al. 2018, 2020; Persad et al. 2019; Lynch et al. 2021) or requirement of non-maleficence and beneficence for an individual subject (Bierer et al. 2021). These principles, supplemented by the principle of respect for autonomy and other considerations regarding non-exploitation, are nevertheless very important. While they do not provide an ethical rationale for offering payment for research participations by themselves, they set contours for an ethically sound payment practice.

## Ethical contours of an ethically sound payment practice

Offering payment for research participants is an incentive *prima facie* required by the principle of the social beneficence. This does not imply, however, that it is ethical to set payment at whatever level necessary and sufficient to attract an adequate number of proper subjects in a timely fashion. On the contrary, no matter how payment is important and effective as an incentive for participation, not every amount, method and timing of payment is acceptable. The reason being the fact that the consequentialistic principle of social beneficence does not exhaust the reining normative framework for human biomedical research. The ethics of biomedical research is built upon a matrix of principles and values which strives to find an adequate balance between the imperative to advance interests of science and society (“research imperative”) and obligations of all societies to protect other important interests of their members, especially interests of research participants and/or involved communities. These latter obligations may be viewed as constituting a general “imperative of non-exploitation” that sets boundaries for the practice of human biomedical research, in general, and for the practice of paying participant, in specific.

### Autonomy and payment

One of the core values behind the imperative of non-exploitation in research is the value of human autonomy. The principle of respect for autonomy is a cornerstone of modern research ethics (National Commission for the Protection of Human Subjects of Biomedical and Behavioral Research 1979; Faden and Beauchamp 1986; Emanuel et al. 2000; Beauchamp and Childress 2001). It is also a bedrock of a fundamental ethical requirement for research, namely informed consent that serves to “ensure not only that individuals control whether or not they enroll in clinical studies”, but also that “they participate only when doing so is consistent with their values and interests” (Emanuel et al. 2000, p. 2706).

The principle of respect for autonomy has a high relevance for the practice of payment for research subjects as one of the most commonly expressed concerns is that payment can be coercive or constitute an undue inducement (undue influence), thus compromising the validity of informed consent (e.g., Macklin 1981; Faden and Beauchamp 1986; McNeill 1997; Dickert and Grady 1999, 2008; Largent et al. 2013; Gelinas et al. 2018, 2020). It is claimed that very high or (for other reasons) overly attractive payment may undermine the capacity of individuals to make autonomous decisions regarding study participation, by compromising their voluntariness, ability to adequate understand and assess research risks and benefits, or by “forcing” them to make choices against their “better judgment or deeply held beliefs” (Council for International Organizations of Medical Sciences 2016, p. 54).

Although there is still a substantial disagreement in the literature about whether an offer of payment may be perceived as coercion, what exactly constitutes undue inducement, and whether money can distort or compromise autonomous decision-making (Macklin 1981, 1989; Wilkinson and Moore 1997; Grady 1999, 2001, 2005; Grant and Sugarman 2004; Emanuel 2004, 2005; Wertheimer and Miller 2008; Klitzman 2013; Largent et al. 2013; Resnik 2015, 2019; Belfrage 2016; Largent and Lynch 2017a, 2017b; Millum and Garnett 2019), existing guidelines and regulatory documents disallow payment, which may unduly seduce people to consent for participation (Largent and Lynch 2017a). Thus, even though overly attractive payment offers could enhance timely recruitment by “alluring” a sufficient number of adequate individuals to join and stay in a study even against their better judgment, there is a regulatory consensus that such payment should not be offered.

The ethical unacceptability of such an overly attractive payment stems from the respect for autonomy. However, it also finds support in the principle of social beneficence. Common sense and empirical evidence, although still limited, suggest that attractive payments may have a negative impact on scientific value of research, as it may encourage potential and/or actual participants to withhold or misrepresent information, which are critical for their recruitment eligibility or continuation in research (Bentley and Thacker 2004; Dresser 2013; Dickert 2013; Devine et al. 2013, 2015; Largent and Lynch 2017a; McManus and Fisher 2018; Lynch et al. 2019)*.* Such a concealment, fabrication or falsification by participants create risks for participants, but also for research resources and the integrity of research data as it can bias the results and undermine the validity of a study (Lee et al. 2018). And—if it occurs frequently enough—it may jeopardize the whole research enterprise. Therefore, assuming—what stills needs to be explored empirically—a positive correlation between the prevalence of deceptive behaviors and the attractiveness of payment (which depends not only on the amount, but also on payment method and timing), the principle of social beneficence provides ethical reasons for employing payment strategies which do not involve overly attractive payment schemes.

The principle of respect for autonomy has fueled much of scholarly discussion on unethical nature of overly attractive payment. However, little attention has been paid to that principle in the context of no-payment and underpayment. Undoubtedly, the lack of resources to cover direct or indirect costs associated with the participation may constitute a barrier for individuals, who otherwise would be willing to take part in research (American Cancer Society Cancer Action Network 2018; Nipp et al. 2019a; Largent and Lynch 2018; Bierer et al. 2021). The removal of these barriers by covering the relevant expenses is important for promoting potential subjects’ autonomy, because it enables individuals to exercise their free will to contribute to the development of science by serving as research subjects. Thus, the principle of respect for autonomy supports paying subjects recompenses, which make the participation in research a cost-free and revenue-neutral activity.

Finally, what is rarely observed, the principle of respect for autonomy provides a general support for public policies which allow remuneration of research subjects for their contributions—both in the form of reward and price—as it calls for respecting people’s right to decide freely in what practices and activates they what to engage in for the sake of earning their living. As Wilkinson and Moore note, “denying people the option of taking inducements reduces their freedom, since it removes an option that they prefer to the alternatives” (Wilkinson and Moore 1997, p. 377). Admittedly, biomedical research enterprise does not aim at broadening the scope of subjects’ freedom or autonomy. Nevertheless, it should not restrict people’s choices without a good reason. Participation in socially valuable biomedical research, whether or not one is paid for it, is neither morally wrong nor bad for the person concerned. On the contrary, it is considered at least as good as engaging in any other socially valuable and risky service or work. Therefore, there are no ethical grounds (either paternalistic or non-paternalistic) for depriving competent individuals an opportunity to serve as research subjects in exchange for money, provided that their decision to participate in a given study is autonomous (i.e., based on comprehensive and adequately understood information, and free from unduly controlling influences).

Moreover, some studies suggest that remuneration can in fact play a positive role in prospective subjects’ consent process by reducing the therapeutic misconception and highlighting research risks. Money might send a message to participants that they take risks and burdens for the sake of the benefit of science and society and “should be compensated for it, which would not occur if they were … expected to benefit from it.” (Glannon 2006, p. 252; also Dickert and Grady 1999; Grady 2001, 2005; Menikoff 2001; Largent and Lynch 2017a). Additionally, some studies suggest that payment can enhance autonomous decision-making by drawing prospective subjects’ attention to research risks and inconveniences (Cryder et al. 2010; Largent and Lynch 2017a; Fisher et al. 2019).

### Justice, fairness and payment

Another ethical principle that has a direct relevance for the practice of paying research subjects is the principle of justice, both in its distributive and commutative dimensions. Many scholars worry that payment may be more attractive to individuals of lower SES, and thus offering payment for participation may result in unfair distribution of research benefits and burdens across the general population (e.g., Maclin 1981, 1989; Faden and Beauchamp 1986; Ackerman 1989; McNeill 1997; Grady 2005; Dickert and Grady 2008; Gelinas et al. 2018). The worse-off will shoulder a disproportionate share of burdens of research, while the benefits will accrue primarily to the better-off. A different, yet linked, concern relates to the risk of exploitation of research participants, especially those of low SES (Lemmens and Elliott 1999; Shamoo and Resnik 2006; Elliott 2008; Elliott and Abadie 2008; Abadie 2010, 2015; Stones and McMillan 2010; Phillips 2011a; Resnik 2015, 2019; Largent and Lynch 2017b; Gelinas et al. 2020; Bierer et al. 2021; MacKay and Walker 2021). Exploitation in research is about the unfair distribution of goods that arise from interaction between researchers and participants (Wertheimer 2008). “One party gets too little, while the other gets too much. Often, but not always, the unfair distribution arises because one party to the interaction is in a weak position, due to poverty, ignorance, or extreme urgency, which the other party can take advantage of, offering few benefits” (Emanuel 2004, p. 101). Economically disadvantaged individuals are in need of money and they tend to value a specific amount of payment more. For many of them accepting an offer of unfairly low but still *en bloc* beneficial payment for participation might be a reasonable choice. After all, even small payment is better than no payment at all, although it constitutes a (mutually beneficial and consensual) exploitation.

These concerns are highly important, but—as it is shown below—they do not exhaust the role of justice/fairness in shaping an ethically sound payment practice. Moreover, in order to fully understand their consequences for ethics of paying research subjects, it is essential to note two things.

Firstly, biomedical research is a social practice embedded in specific social reality shaped by its historical and cultural roots, reigning power- and economic relations, and normative fabrics. This social reality is marked by profound social and economic inequities (within concrete societies as well as at the level of international community), which should be mitigated, and ideally eliminated by broad social reforms. Research enterprise should not entrench or exacerbate these background social injustices. However, it is neither designed for nor capable of actively fighting them, especially against participants’ poverty, unemployment or lack of access to high-quality healthcare (Fisher 2019). Justice in research requires treating all research participants fairly and equitably, also when it comes to payment for their participation. But it does not require paying participants of low SES in order to alleviate or remove hardships of their position. Such a positive impact of research payment on subjects’ social or economic condition is laudable, and even desirable, but it is not a demand of justice.

Moreover, contrary to views of some commentators, the commutative justice per se does not require offering payments to research subjects. Largent, Emanuel and Lynch claim that “when goods and services are not indented as gifts, failure to pay for them is a problem: we call it theft” (2019, p. 1), thus suggesting that this is exactly what happens when participants are not fairly paid for their contribution to the common good. Despite its rhetorical attractiveness, this claim cannot be accepted as it rests on two mistakes. Firstly, it ignores the power of consent. Valid consent of a prospective subject for using her body for research purposes transforms theft into gift, lease, rent, work or other consensual relation with a researcher. Second, it forgets that it takes two willing parties to change provision of goods or services into transaction, i.e., exchange of goods and services in return for money. Thus, when a researcher is unable (e.g., due to budgetary constraints) or uninterested in offering payment for prospective participants (e.g., due to availability of sufficient number of unpaid subjects), or when a prospective participant is genuinely willing to contribute to the development of science without any remuneration and reimbursement, there is no ground for transaction. And there is nothing essentially unfair in allowing researchers and altruistically motivated participants to engage in scientifically and socially valuable biomedical research.

Payment is a recruitment incentive justified and *prima facie* required by social beneficence, not by justice or fairness. However, *when it is to be offered*, the offer should satisfy the requirement of distributive and commutative justice.

It is commonly accepted that the principle of justice requires distributing burdens and benefits of study participation in such a way that no segment of the population is unduly burdened by research or denied its potential or actual benefits. Recruitment criteria should reflect the scientific purpose of the study, not target populations which are considered “easy to recruit” “simply because of their easy availability, their compromised position, or their manipulability” (National Commission for the Protection of Human Subjects of Biomedical and Behavioral Research. 1979). Thus, researchers must neither exploit the vulnerable, in particular economically disadvantaged, nor exclude without good reason those who stand to benefit from study participation. They should strive to recruit an adequate cross-section of the population in order to spread research burdens and benefits fairly across the population. In other words, an adequate diversity of gender, race, ethnicity, age and SES in research should be sought (Geller et al. 2011; Kwiatkowski et al. 2013; Heller et al. 2014; Winter et al. 2018; cf. Dickert 2009; Bierer et al. 2021). It is worth noting that the last postulate finds an additional support in the principle of social beneficence as it enables the generalization of knowledge to be gained in research, thereby enhancing research scientific and social value (Wilkinson and Moore 1997; Grady 2005; Resnik 2015; Largent and Lynch 2017b). Thus, justice encourages payment schemes which have a potential of making distribution of research burdens fairer. And justice is against schemes that can deepen social inequalities, lead to unfair social distribution of research burdens and benefits or exploitation.

What should research subjects be paid for and in which amount to make payment consistent with demands of justice? Firstly, justice-related considerations provide an ethical justification for paying research subjects a recompense for direct costs related to research participation. By making participation in research a cost-free activity, recompense removes—at least some—economic barriers for participation, thus equalizing opportunities for all willing individuals to contribute to the development of science, no matter their SES. Moreover, as studies already referred to suggest (Unger et al. 2013, 2016, 2019; American Cancer Society Cancer Action Network 2018; Nipp et al. 2019b; Chino and Zafar 2019), reimbursement of direct expenses may improve access to potentially beneficial studies for patients, who otherwise would not be able to participate due to their low SES or disease-caused greater sensitivity to economic burdens associated with research. Thus, the reimbursement of direct costs has a potential to enhance justice in research not only by reducing inequities in access to research for those who otherwise could not afford it, but also by contributing to the fair distribution of clinical benefits associated with participation.

Secondly, the principle of justice provides a strong ethical reason for disallowing recompenses for the loss of reasonably expected profits, especially lost wages. Such recompenses lead to differential payments between participants—individuals who receive higher wages get higher recompense; those who are lower paid by employees—lower recompense, and those who are unemployed—no recompense at all. This is consistent with a norm of equity, as it makes participation a revenue-neutral activity for all, no matter how much they earn. Nevertheless, it is very likely to reinforce unfair distribution of research risks and benefits between different social strata by prompting researchers to make savings by drawing research participants from “cheaper” populations, especially from the unemployed and the low-paid (Dickert and Grady 1999; Resnik 2015).

Thirdly, justice supports remuneration for participants’ contributions to the development of science and society, provided it is fair, i.e., equitable, adequate, and non-exploitive. Remuneration is equitable when it does not violate the norm “equal pay for equal work” (Dickert and Grady 1999; Resnik 2015; Gelinas et al. 2018, 2020; Persad et al. 2019). Equal time, efforts, burdens and risks associated with participation deserve equal remuneration (measured in market value, rather than in nominal value in case of multi-site studies conducted in different settings). However, when different groups of participants in a study are expected to make different contributions, equitable remuneration should reflect differences in their input (Persad et al. 2019). This is consistent with equal respect and concern for each and every individual and it prevents discrimination.

Remuneration is adequate when it is proportionate in value to the value of participants’ contribution to the study. The latter necessarily depends on various factors which determine how time- and effort-consuming, burdensome and risky give research project is. For example, when a study involves 2 visits, each lasting 60-min and requires filling a questionnaire and giving a blood sample for further analysis, and another project involves 4 visits of the same length and level of associated risks and burdens, participants of the latter study—*ceteris paribus*—should be offered remuneration of double the remuneration offered to the participant of the first project.

All guidelines and regulatory documents referred to above follow this normative logic by recommending or permitting paying research participants an “appropriate” or “proportionate” or “just and fair” remuneration for the time spent and other inconveniences resulting from the study participation (Council of Europe 2005b, sec. 64; Health Research Authority 2014, par. 3.4; Council for International Organizations of Medical Sciences 2016, p. 53; Food and Drug Administration 2018; National Health and Medical Research Council 2019, par. 1.1). Only a few, however, mention the level of research risks among factors which should be taken into consideration when determining an adequate amount of payment. Moreover, those that do so provide conflicting instructions on this matter. For example, the CIOMS Guidelines *expressis verbis* state that “the level of compensation should not be related to the level of risk that participants agree to undertake” (2016, Commentary on Guideline 13). In contract, the Australian National Health and Medical Research Council’s guideline reads: “In cases where risk may be considered as a factor in determining payment, payment of participants based on the degree of risk associated with the research is not prohibited, so long as there is evidence that a participant’s ability to provide valid consent is not likely to be compromised” (2019, par. 1.3.). Also the UK Health Research Authority “sympathises with the view that not to allow payments on the basis of risk would be unduly paternalistic in the absence of evidence that the participants’ ability to provide valid consent would be compromised” (2014, par. 3.1.).

These regulatory variations mirror the lack of consensus regarding risk-based payments among research ethicists (Grady 2001; Menikoff 2001; Jones and Liddell 2009; Saunders 2009; Grimwade et al. 2020; Lynch and Largent 2020; Jamrozik and Selgelid 2020). Although a detailed analysis of this issue goes beyond the scope of this analysis, three following arguments provide strong reasons for arguing that adequate remuneration for research subjects should be proportionate also to the level of risk involved in participation: (i) an argument from consistency, which notices that risk-based remuneration is accepted in many non-research contexts, e.g., in high-risk professions (Menikoff 2001; Jones and Liddell 2009); (ii) an argument from the nature of human “guinea pigging” which claims that the assumption of risk is often an essential contribution of research participants (Menikoff 2001; Różyńska 2018; Malmqvist 2019); (iii) an argument for public trust, which argues that paying proportionally to the incurred risk meets expectations regarding the fair treatment of a significant portion of prospective subjects as well as researchers, REC/IRB members, and other members of the research community (Czarny et al. 2010; Ripley et al. 2010; Largent et al. 2012; Grimwade et al. 2020), thereby enhances public trust in research at large.

Finally, remuneration is non-exploitive when it is not lower than a socially accepted payment which is (or would be) offered for a similarly time- and effort-consuming, burdensome and risky activity, outside research context in the same setting (Gelinas et al. 2018; Largent and Lynch 2017b). Although space does not permit discussing in details how a proper amount of such defined payment should be calculated, a proposal widely advocated in the literature should be mentioned here, namely a minimum hourly wage benchmark. Numerous authors and guidelines suggest that the amount should be based on the minimum hourly wage in the region or country as a point of reference (Council for International Organizations of Medical Sciences 2016, Commentary to Guideline 15, 53; National Health and Medical Research Council 2019, Appendix 1, 7) with augmentations for particularly burdensome procedures (Ackerman 1989; Dickert and Grady 1999; Grady 2005; Gelinas et al. 2020), risks involved (Menikoff 2001), and even other additional benefits (cf. Anderson and Weijer 2002). This proposal has three advantages. It is relatively easy to implement as it provides a clear method of setting the baseline amount of reimbursement for research participants. It allows for keeping payments sensitive to specific features of the project and subjects’ contributions by accepting relevant payment augmentations. And—since it sets the reference value of an non-exploitive remuneration relatively low—“more researchers could afford to pay a fair wage and fewer would be inconvenienced by a prohibition on unfairly low wages” (Phillips 2011a, p. 219). However, it also faces challenges. Offers of payment calculated in such a way would be attractive for prospective subjects with low SES, who have no reasonable alternatives to get engaged in better paid activities. But these offers would likely have no impact on recruitment of individuals who are better-off, i.e., who have better paid jobs or capacity to make more money outside research context, thus potentially biasing the subjects’ recruitment. In contrast, the average-wage benchmark for non-exploitive remuneration, mentioned by some authors (Phillips 2011a) would seem fairer as it would make payment offers reasonably attractive to both individuals of SES and to those better-off, thus promoting a fair distribution of research risks and benefits between different social strata.

Fourthly, the principle of justice does not prohibit remunerations going beyond what constitutes equitable, adequate, and minimally non-exploitive payment—i.e., remunerations driven by a market-driven forces. Although such payments are conceptualized as “price” for desired services, in order to avoid exploitation, they should not be lower than remuneration viewed as “reward”. They may, however, be higher than “rewards” and they may vary within the same study on the basis of salient characteristics of particular groups of participants (i.e., their age, sex, race, ethnicity, rare clinical status etc.). Offering disproportionally high and/or differential remuneration does not violate requirements of justice insofar as the payment is designed to help the study to meet its social and scientific goals by enhancing recruitment and retention of the necessary category of subjects, and it does not reproduce or reinforce wider social inequities and injustices, e.g., racial biases or class differences (Persad et al. 2019).

### Individual beneficence and payment

The principle of individual beneficence is rarely invoked in the discussion on ethical payment practices. Most probably, it is because research is about advancing the interests of science and society, not the interests of individual participants (Emanuel et al. 2000; Miller and Brody 2003).While there are studies which have a potential of direct therapeutic benefits for the participants, it is neither the goal of research practice, nor a requirement that research should be beneficial for participants. Moreover, all canonical guidelines on human research ethics exclude non-direct and non-medical benefits to research subjects, such as payment, from the risk–benefit analysis, thereby forbidding IRBs/RECs to take payment into account as a benefit to counterbalance research risks (Emanuel et al. 2010; *contra* Wertheimer 2013).

Nevertheless, despite the above normative premises, in fact, individuals treat money as a benefit when considering an offer to participate, and deciding on entering the study (Abadie 2010; Czarny et al., 2010; Stunkel and Grady 2011; Grady et al. 2017; Fisher et al. 2018; Manton et al. 2019). For an individual prospective participant, the payment is a part of an equation for the overall attractiveness of a research project. Recompenses, when full and adequate, make the research participation cost-free for subjects, and—as shown above—they can remove at least some entrance barriers for patients to potentially clinically beneficial studies. Remunerations—whether calculated as a reward or as a price—may constitute a gain for subjects, thereby making participation in research overall beneficial from their personal perspective.

Thus, the principle of individual beneficence provides additional support for offering money to research subjects. However, the same principle justifies the claim that payment for participation should not be overly attractive. There is some evidence that too attractive remuneration may increase the risk of jeopardizing subjects’ health by encouraging them to conceal or misrepresent information important for their safety in order to ensure recruitment or continued participation in paid research (Bentley and Thacker 2004; Devine et al. 2013, 2015; Lee et al. 2018; Lynch et al. 2019). Such a deceptive behavior by participants may take various forms (e.g., nondisclosure of concurrent enrollment in other studies, concealment of tobacco use, alcohol consumption, or illicit substance abuse, concealment of pre-existing medical conditions, falsification of current health status, over-reporting of a study protocol adherence, etc.), and it may result in severe adverse events or even subjects’ death (Lee et al. 2018).

## Conclusions

The paper argues that the ethical anatomy of paying research participants is built upon four basic principles of research ethics (and bioethics in general). The ethical “spine” of the practice is the principle of social beneficence, which requires the maximization of the common good—in the case of research practice—socially valuable scientific knowledge. This principle grounds a general *prima facie* moral obligation of offering payment to research subjects. The remaining ethical principles constitute a “skeleton” of morally sound payment practices by providing additional moral reasons for offering or not offering certain types of payments to research participants. As discussed above and presented in Table 1, the principles argue for offering research participants:

- recompense for reasonable expenses, but not for lost wages (or loss of other reasonably expected profits);
- remuneration conceptualized as a reward for their valuable contribution, provided (i) the remuneration meets standards of equality, adequacy and non-exploitation, and (ii) it is not overly attractive, i.e., it does not constitute undue inducement for participation or retention, and does not encourage deceptive behaviors;
- remuneration conceptualized as a market-driven price, provided (i) the remuneration is necessary and designed to help the study achieve its social and scientific goals, (ii) it does not reinforce wider social injustices and inequalities; (iii) it meets the requirement of non-exploitation; (iv) it is not overly attractive (as defined above).

**Table 1:**
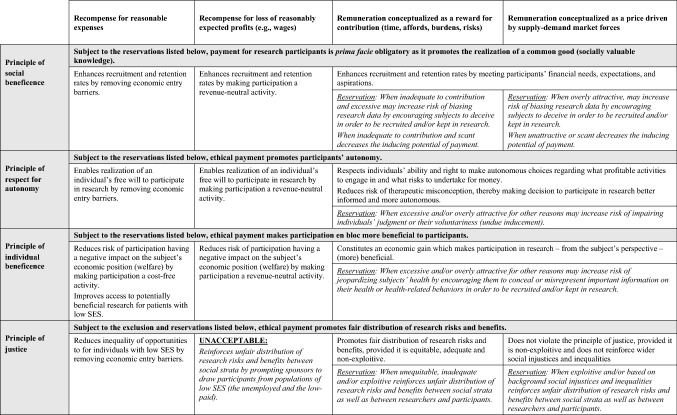
The ethical anatomy of payment for research participants

Obviously, a proper application of this ethical “skeleton” into research practice requires investigators and RECs/IRBs to take into account “the nature of the study, the nature of participants contributions and vulnerabilities, institutional and organizational guidelines, and local and cultural norms” (Grady 2005, p. 1686). Moreover, to make the proposed scheme fully helpful in determining whether any particular offer of payment is not overtly attractive and whether it meets standards of not equality, adequacy and non-exploitation, further detailed analyses of these standards are needed. The proposed scheme should also be tested against and enriched by further empirical studies about payment, especially about how money impacts subjects’ decision-making processes and behaviors.

## Data Availability

Not applicable.

## Footnotes

1 This is an improved version of the payment typology presented in Różyńska (2021).

2 Developing an appropriate understanding of a common good, also referred to as a public, social or collective good, is a challenge that cannot be addressed here. For the sake of this analysis, I use all these terms interchangeably, as is frequently done in the literature. My understanding of a common good is based on the conceptual analysis provided by Hans Radder (2017). It is also inspired by Alex London’s idea of “the generic interests conception of the common good” by assuming that a social practice or policy is socially valuable when it overall promotes “a subset of interests that are sufficiently basic or fundamental to individuals that they are common to each of the members of the community… interests in being able to develop their intellectual and affective capacities in order to pursue activities that they find meaningful, and to engage in meaningful relationships with others” (2003, p. 21).
